# People accurately predict the transition probabilities between actions

**DOI:** 10.1126/sciadv.abd4995

**Published:** 2021-02-26

**Authors:** Mark A. Thornton, Diana I. Tamir

**Affiliations:** 1 Department of Psychological and Brain Sciences, Dartmouth College, Hanover, NH 03755, USA.; 2 Department of Psychology and Princeton Neuroscience Institute, Princeton University, Princeton, NJ 08540, USA.

## Abstract

Social life is a complex dance. To coordinate gracefully with one’s partners, one must predict their actions. Here, we investigated how people predict others’ actions. We hypothesized that people can accurately predict others’ future actions based on knowledge of their current actions, coupled with knowledge of action transitions. To test whether people have accurate knowledge of the transition probabilities between actions, we compared actual rates of action transitions—calculated from four large naturalistic datasets—to participants’ ratings of the transition probabilities between corresponding sets of actions. In five preregistered studies, participants demonstrated accurate mental models of action transitions. Furthermore, we found that people drew upon conceptual knowledge of actions—described by the six-dimensional ACT-FASTaxonomy—to guide their accurate predictions. Together, these results indicate that people can accurately anticipate other people’s moves in the dance of social life and that the structure of action knowledge may be tailored to making these predictions.

## Teaser

People accurately predict sequences of everyday actions, supported in part by conceptual knowledge tailored for such predictions.

## INTRODUCTION

Social interactions are a complex dance. To coordinate gracefully with one’s partner, one must read their moves and anticipate their next steps (*1*). Failing to predict others’ moves could lead to a life of perpetually stepping on others’ toes, or vice versa (*2*). As intricate as dancing the tango may be, the full repertoire of human behavior is far richer than the steps that unfold on any dance floor (*3*). People engage in an astonishingly wide array of different actions and activities. These actions run the gamut from concrete physical procedures, like hammering a nail, to abstract social processes, like building consensus. Despite the rich array of actions that humans can perform, people are remarkably successful at navigating the dance of social life. How do people so accurately predict others’ future actions?

Researchers have identified at least three sources of information that people can use to make action predictions. First, people use environmental cues to predict which actions are possible, and which are most likely. That is, people observe the objects in an environment (*4*–*7*), or other physical features of the space, to determine what action affordances they offer (*8*, *9*). For example, people are more likely to sit in a room containing chairs and more likely to stand in a room that lacks them. Second, people use perceptual cues about other’s actions to predict what they will do next, both by attending to others’ biological motion (*10*) and by simulating others’ motor activity (*11*). For example, if you see someone walking with a hurried gait, you might infer that they are more likely to be rushing to the bathroom than just out for a stroll. Likewise, if you wanted to predict how fast your teammate will run in the next play of a sports game, you could imagine how tired you would feel if you ran like they did in the last play (*12*, *13*). Third, people use cues about others’ latent mental state cues to predict their actions. Goals, intentions, and emotions are excellent predictors of future actions: If you know someone wants a sandwich, you can predict that they will soon act to obtain one. Knowledge of someone’s intentions or goals can also help prioritize the collection of additional perceptual information (*14*–*16*). For example, if you think someone is thirsty, your eyes may automatically dart to the water jug to see whether they will reach for it. Emotions too are often a useful clue to how people will behave (*17*): Angry people aggress, tired people rest, joyful people sing and dance, and so forth. On even longer time scales, people’s habits and personality can inform action predictions because these traits effectively describe the base rates of certain behaviors (*17*, *18*). If a friend habitually orders the lentil salad at your favorite lunch spot, you can guess that they will probably do it again the next time you eat there. Similarly, if a friend is highly extraverted, you can accurately predict that they will come to your next house party.

We propose that, in addition to these well-studied action prediction cues, people also rely heavily on a fourth, understudied, source of information to make action predictions: other actions. A person’s current action says a great deal about their likely future actions. This is because actions do not usually occur in random succession. One is much more likely to see someone buy groceries, cook a meal, and then eat it, than to see these actions play out in reverse order. Sequences such as this one produce regularities in the transition probabilities between actions. Transition probabilities represent the likelihood of a future action, given one’s current action. Sometimes transition probabilities are high—such as between cooking and eating—and other times they are low—such as between eating and swimming. Regularities in transition probabilities result both from strictly linear sequences of actions and from clusters of actions that tend to happen close together in time, but without any particular order. For example, running, walking, stretching, and drinking water tend to happen close together in time, but not necessarily in any one order.

Regularities in action transitions have been studied extensively in nonhuman animals such as fruit flies (*19*). Even in creatures with comparatively simple nervous systems and behavioral repertoires, the examination of action transitions has revealed remarkably sophisticated structure, with hierarchical organization across multiple time scales. Studies of human action dynamics have primarily focused on the dynamics within actions, such as how the motor control of reaching actions unfolds over time (*20*). Studies focusing on transitions between actions have found complex dynamical processes governing how the human body moves from one mode to another (*21*). Together, this research suggests that people act in predictable sequences, and a savvy perceiver could use these regularities to make action predictions.

There is reason to believe that perceivers do use these regularities to make predictions. People have a well-developed capacity for statistical learning. Early research in this domain examined how statistical regularities in language influenced word acquisition. This research found that infants are capable of parsing a speech stream by detecting regularities in the transition probability structure of nonsense words (*22*). Subsequent studies in adults have extended these findings to other domains—such as vision (*23*)—indicating that people have a robust capacity to learn the statistical regularities in their environments. Recent findings indicate that people apply this capacity to social prediction as well. For instance, people have highly accurate mental models of the transition probabilities between different emotions (*24*). Thus, people seem to have the prerequisites to learn the statistical regularities between actions (*25*).

Most studies that have tested whether people can predict action transitions have focused on infants and toddlers (*26*–*28*). Even at this young age, children seem able to deploy this ability automatically, anticipating predictable action sequences with their gaze. For example, infants appear more surprised when someone pauses in the middle of an action, than when they pause after having completed it. This ability to predict actions based on statistical learning persists into adulthood, and also grows more flexible, as adults learn to modify their action predictions based on context (*29*). For example, adults who performed well on a measure of statistical learning were especially accurate in predicting upcoming actions when the agents performing those actions shared a common goal. These previous studies have established the ability of adults and children to predict action transitions. That said, both psychological and neural evidence point to the existence of critical or sensitive periods during childhood for learning about domains such as language (*30*, *31*). Potential differences between children and adults highlight the importance of expanding the small literature on adult prediction of action transitions.

Previous studies in this vein have either probed participants with small numbers of naturalistic action sequences or artificially manipulated the transitions between actions. Although these paradigms offer experimental control, they also bring limitations. Working with small numbers of naturalistic transitions inherently limits the generalizability of findings because it is unclear how representative the sampled transitions are of the broader domain. Similarly, artificially manipulating action transitions limits ecological validity. The ability to learn artificial action transitions does suggest that people might be able to do the same with natural transitions. However, the artificial transition structures in such studies may not resemble the structure of naturally occurring action transitions. Certain transition structures could be easier or harder to learn for a variety of reasons, such as memory interference. Together, these limitations emphasize the need to study large, naturalistic sets of action transitions.

The primary goal of the present study is to test whether people can accurately predict action transitions across the rich repertoire of natural human behavior. To do so, we first measure actual action transitions using five naturalistic datasets containing action sequences, including movie scripts, day-recall surveys, written instructions, and annotated videos. These datasets cover a broad spectrum of actions, varying in time scale from seconds to hours, varying in sources from text to video, and varying in abstraction from precise motor actions to broad activity categories. We use these five datasets to estimate the “ground truth” probabilities of how actions actually transition from one to the next. In five preregistered studies, we then compare these ground truth measures of transition probabilities to people’s predictions of the same transition probabilities. In each study, participants rate the likelihood of a future action, given a current action. For example, “how likely is someone to start running, given that they are currently stretching?” or “how likely is some to start dancing, given that they are currently laughing?” We measure the accuracy of participants’ judgments by correlating their ratings with the ground truth measures of the same transitions.

After we determine whether people can make accurate action predictions, our second goal is to determine how people make accurate predictions. We have previously proposed a theoretical framework for predictive social cognition, which suggests that social knowledge is organized by the goal of prediction (*32*). That is, the way in which people organize their conceptual knowledge of actions should allow them to make accurate action predictions. In previous research, we found that six psychological dimensions—together forming the Abstraction, Creation, Tradition, Food, Animacy, and Spiritualism Taxonomy (ACT-FAST; Table 1)—capture much of people’s conceptual knowledge of actions (*33*). Each action can be located on this “map” of actions based on its relation to each dimension. For example, the actions “stir” and “peel” load high on the food dimension; the action “dance” loads low on the food dimension, but high on the creation and animacy dimensions. Actions located closer to each other on these dimensions are more conceptually similar to each other. The ACT-FAST dimensions also describe how people infer the real-world properties of actions (e.g., where, why, when, how, and by whom different actions are performed) and predict patterns of brain activity elicited by perceiving actions (*33*, *34*).

**Table 1:** Dimensions of ACT-FAST.

**Dimension**	**Pole 1**	**Pole 2**	**Examples**
Abstraction	Abstract/social	Concrete/ physical	Govern, refute vs. drip, peel
Creation	Creation	Crime	Film, sing vs. prosecute, testify
Tradition	Tradition	Innovation	Cook, decorate vs. emit, encrypt
Food	Food	Nonfood	Bake, fry vs. detain, testify
Animacy	Animate	Mechanical	Meow, floss vs. contain, extract
Spiritualism	Work	Worship	Fax, haggle vs. foretell, ascend

According to our predictive social cognition framework, actions located closer to each other on the ACT-FAST dimensions are also likely to have high transition probabilities between them. That is, proximity on these dimensions does more than just describe patterns of conceptual similarity; they also describe transition likelihoods (*24*). For example, if another person is currently peeling vegetables, one could accurately predict that they are more likely to stir a pot in the near future than they are to dance around the vegetables. That is, peeling vegetables and stirring a pot are not only conceptually similar; they also tend to happen close together in time (e.g., during meal preparation). Thus, ACT-FAST facilitates action prediction by organizing conceptual knowledge about actions along these six temporally predictive dimensions.

Not all possible dimensions of action representation have this temporally predictive property. For example, consider representing actions in terms of which body parts they rely on. Actions that rely on different body parts are seamlessly intermixed in the course of everyday experience. Imagine you are standing at a street corner waiting to meet a friend for lunch. You feel your phone buzz and automatically reach in your pocket to retrieve it (using your hands). You read a text from your friend (using your eyes) that suggests you meet them at a coffee shop right around the corner. Prompted by this message, you start walking to meet them (using your legs). This sequence of actions can be easily explained in terms of their highly social purpose: They are all oriented toward the goal of meeting your friend. However, one could not predict which actions would follow which based on the body parts involved in executing them. Tacking the body parts involved in action does have practical value—just not for predicting action transitions. Instead, some action features may simply be more useful for prediction than others.

We hypothesize that the ACT-FAST dimensions represent the aspects of action concepts that serve a specifically predictive purpose. We examine this hypothesis here by measuring the positions of actions on the ACT-FAST dimensions, and then testing whether the proximity between them statistically mediates the accuracy of participants’ action predictions.

## RESULTS

### Accuracy of action prediction

**Fig. 1:**
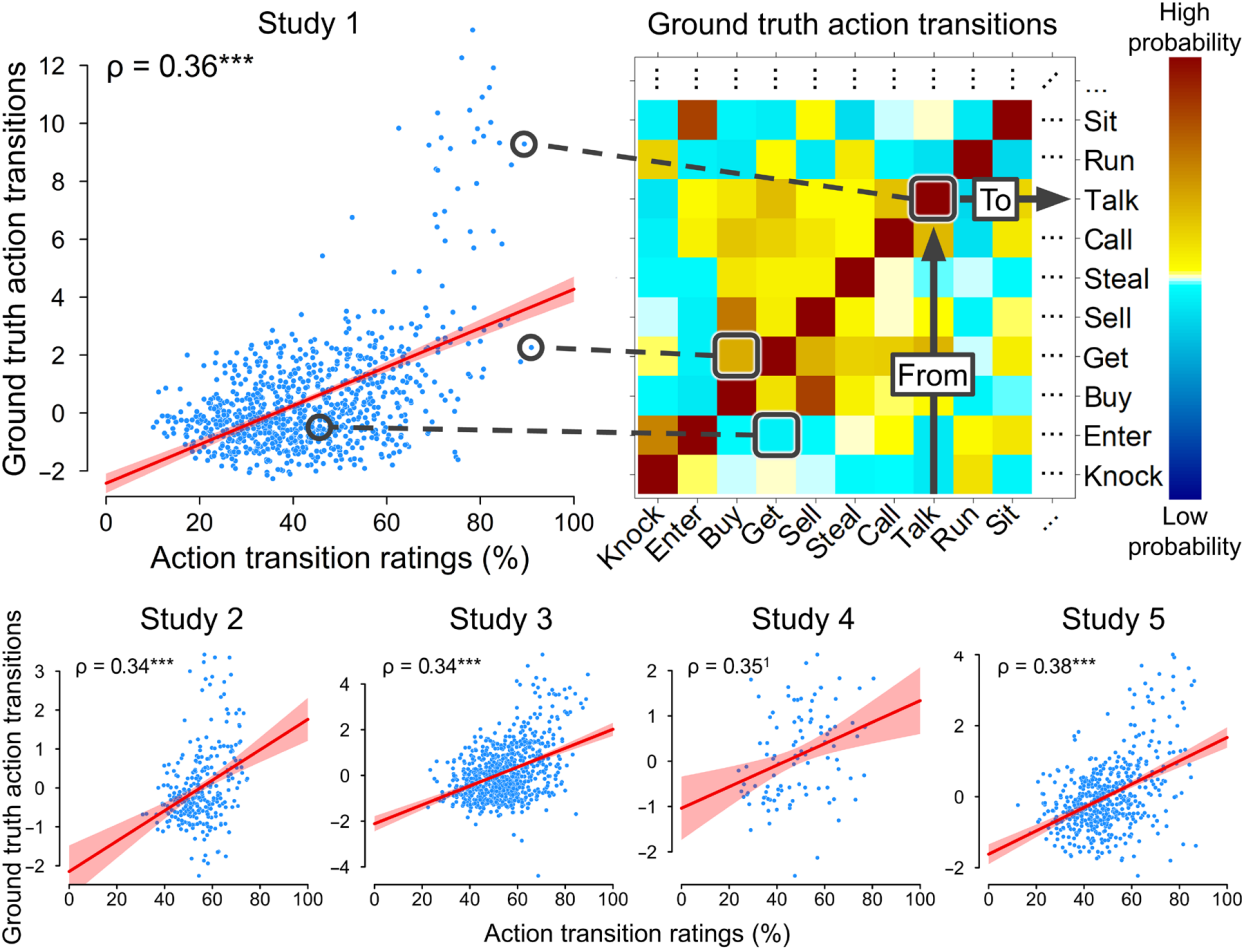
Action transition ratings accurately predict ground truth. The heatmap illustrates a subset of the ground truth action transition probabilities for study 1. Scatter plots illustrate the association between these ground truth transition probabilities (*z*-scored log odds; *y* axis) and the rated transition probabilities for each pair of actions (*x* axis), averaged across participants. All studies showed significant associations between rated and actual transitions, assessed using Spearman rank correlations, ρ (***P < 0.0001). The scatterplots show linear best fit lines and parametric confidence intervals (CIs) (red) for illustrative purposes. ^1^Preregistered inferential tests in study 4 were conducted only at the individual participant level.

Our primary goal was to assess whether people accurately predict the transition probabilities between real-world actions. We first measured the actual transition probabilities between actions to serve as a “ground truth” against which to compare people’s perceptions. We computed these ground truth transition probabilities using five different datasets. In study 1, we analyzed actions in movies, using movie scripts from IMSDb.com. We used verbs as a proxy for actions and estimated action transitions as verb-to-verb transitions across lines of spoken dialog and stage direction (Fig. 1). In studies 2 and 3, we analyzed actions that people self-reported engaging in over the course of the preceding day as part of the American Time Use Survey (ATUS) 2003–2016 (*35*). The ATUS asks people to list their sequence of actions over the course of a day by choosing from menus of activities. Each action must be specified at both a broad level (e.g., “personal care” and “household activities”) and a narrow level (e.g., “sleeping” and “eating”). We examined transitions at the broad and narrow level of the ATUS in studies 2 and 3, respectively. In study 4, we analyzed sequences of actions in sets of instructions from WikiHow.com. We estimated action transitions as verb-to-verb transitions across steps in each set of instructions. Last, in study 5, we analyzed actions in video from Google’s Atomic Video Actions (AVA) dataset. Together, these five datasets offer a breadth of naturalistic action sequences, collected across multiple modalities, time scales, and sources, to serve as our ground truth action transitions.

We next measured people’s predictions of the same transitions. Participants rated the transition probabilities between actions in each of the five ground truth datasets. For example, a participant in study 1 might see a prompt that read “to run → to jump” with instructions reading “If someone is currently engaged in the action on the left, how likely is it that they (or someone else) will next engage in the action on the right?” Participants made their responses on continuous line scales ranging from “0% (Never)” to “100% (Always).” To estimate the accuracy of these ratings, we correlated them with the corresponding ground truth estimates. This analysis was performed at two different levels of analysis.

First, we tested the accuracy of ratings for each action transition, averaged across all participants. In this item-level analysis, we averaged participants’ ratings for each action transition to produce a single transition probability estimate for that item. We correlated the set of average ratings with the set of ground truth to estimate accuracy. This item analysis was performed for all studies except study 4 (in study 4, participants did not rate a full transition matrix, but only selected elements; all analyses were done at the individual level). We observed a significant association between rated and ground truth transition probabilities in all four studies, with correlations all between ρ = 0.34 and ρ = 0.38 (Fig. 1 and Table 2). These results indicate that people can accurately predict action transitions.

**Table 2:** Item-level accuracy effects and analysis variants.

**Study**	**Full matrix**		**No diagonal**		**Symmetric**		**Asymmetric**		Maximum*
	**ρ**	***P***	**ρ**	***P***	**ρ**	***P***	**ρ**	***P***	**ρ**
1	0.36	<0.001	0.30	<0.001	0.40	<0.001	0.14	0.0025	0.65
2	0.34	<0.001	0.31	<0.001	0.38	<0.001	0.20	0.0020	0.86
3	0.34	<0.001	0.30	<0.001	0.38	<0.001	0.15	0.0085	0.64
4	0.35^†^								
5	0.38	<0.001	0.31	<0.001	0.43	<0.001	0.09	0.060	0.72

Note: Coefficients reflect the results of item analyses, in which ratings were averaged across participants before correlating them with ground truth transition probabilities. All correlations were computed using Spearman’s ρ. Full matrix refers to correlations with the entire ground truth transition probability matrix. No diagonal refers to the same correlation but with the diagonal removed. Symmetric and asymmetric refer to correlations between only the symmetric or only the asymmetric off-diagonal components of the transition probability matrices. In all cases, *P* values were calculated using the Mantel test.

*Expectations for maximum expected correlations were calculated by combining the reliabilities of the ground truth and rating matrices for each study using Spearman’s method for correlation disattenuation.

†Inferential item analyses were not conducted in study 4, as we selected an optimized subset of action transitions. We provide the correlation coefficient across all transitions included in that study for descriptive purposes.

We conducted three additional variants of this item analysis to assess which components of the transition matrix supported participants’ accuracy. Specifically, we examined whether people could accurately report the (i) off-diagonal, (ii) symmetric, and (iii) asymmetric components of the transition probability matrices. Results suggest that people can predict autocorrelated actions, temporally clustered actions, and common action sequences, respectively, but do not rely on any one of these alone to achieve their overall accuracy (Table 2).

For each study except study 4, we also computed the maximum item-level correlation we could expect to observe given the reliability of the data (i.e., a “noise ceiling”). These values (Table 2) give greater context to the item-level accuracy correlations that we observe in studies 1 to 3 and 5. Specifically, we calculate that participants achieved 55, 40, 53, and 53% of ceiling accuracy in each of these studies, respectively. Chance level performance was also estimated for each analysis variant in each study (table S1).

**Fig. 2:**
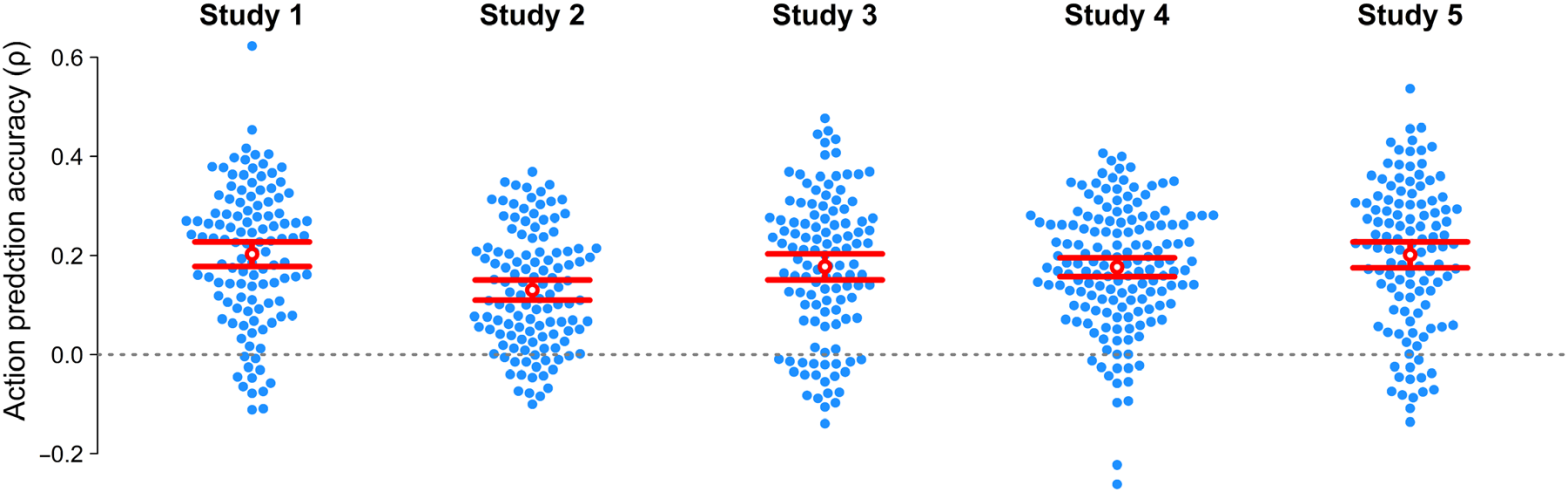
Individual participants’ transition ratings accurately predict ground truth. Each point represents the correlation between one participant’s transition probability ratings and that study’s ground truth estimates. Bee swarm plots indicate the distribution of accuracy across participants. In all five studies, most individual participants made accurate predictions, with the average (red) significantly (95% CIs shown) above chance (gray) accuracy.

In addition to testing the accuracy of transition ratings averaged across participants, we also the tested accuracy of each individual participant. In all five studies, we observed that the average correlation between each participant’s ratings and ground truth significantly was greater than zero, with means ranging from ρ = 0.13 to 0.20 (Fig. 2 and Table 3). These results provide further evidence that people can accurately predict action transitions.

**Table 3:** Individual-level accuracy effects.

**Study**	***N***	**Mean ρ**	***P***	**95% CI**	***d***
1	116	0.20	1.8 × 10^−29^	[0.18, 0.23]	1.42
2	121	0.13	1.2 × 10^−22^	[0.11, 0.15]	1.10
3	117	0.18	1.8 × 10^−24^	[0.15, 0.20]	1.21
4	156	0.18	1.5 × 10^−39^	[0.16, 0.20]	1.43
5	117	0.20	6.4 × 10^−28^	[0.17, 0.23]	1.35

Note: Accuracy was computed separately for each participant as the Spearman’s ρ correlation between a participant’s transition ratings and ground truth. *P* values reflect one-sample *t* tests on Fisher *z*-transformed correlations. Ninety-five percent confidence interval (CI) values reflect percentile bootstrap CIs on untransformed ρ values. Cohen’s *d* values were computed by dividing the mean *z*-transformed ρ by the corresponding SDs.

In study 2, the small size of the transition matrix allowed us to assess the four variants of the item analysis at the individual level too. We observed that individual participants’ ratings accurately reflected all four components of the transition probability matrix: the full matrix (Table 3), the off-diagonal component [mean ρ = 0.12, *P* = 9.9 × 10^−24^, 95% confidence interval (CI) = [0.11, 0.14], *d* = 1.15], the symmetric component (mean ρ = 0.16, *P* = 2.0 × 10^−23^, 95% CI = [0.13, 0.18], *d* = 1.13), and the asymmetric component (mean ρ = 0.05, *P* = 5.4 × 10^−10^, 95% CI = [0.037, 0.067], *d* = 0.61). These outcomes provide further evidence of the robust and nuanced nature of participants’ insight into action transitions.

### Conceptual mediators of action prediction

People have accurate intuitions about the transition probabilities between actions. How do people achieve this accuracy? Here, we considered the hypothesis that people organize their conceptual knowledge of actions in a manner that facilitates prediction. Specifically, we predicted that the proximity between actions on the dimensions of the ACT-FASTaxonomy (Table 1) would correlate with both perceived and actual transition probabilities and statistically mediate the association between them. To test this hypothesis, participants rated where each action from studies 1 to 5 placed on each ACT-FAST dimension. We then computed the proximities between each pair of actions on each dimension and regressed these ratings out of both the perceived and actual transition probabilities. Last, we correlated the residual perceived and actual transition probabilities with each other, as we previously did in the item-level accuracy analyses. We compared these partial correlations with the corresponding full correlations. If the partial correlation was significantly less than the full correlation, this would indicate that ACT-FAST accounts for shared variance between perceived and actual transition probabilities.

**Fig. 3:**
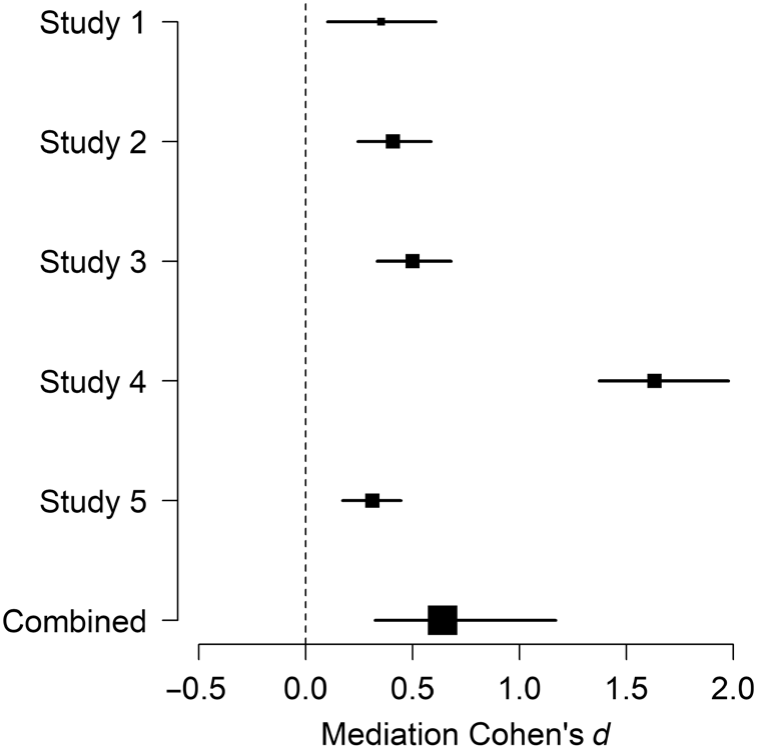
The structure of action knowledge scaffolds accurate action prediction. People can accurately predict how actions transition from one to the next. A mediation analysis shows that people make action predictions, in part, via the ACT-FAST. In all five studies, proximity on the ACT-FAST dimensions significantly explains the association between rated and ground truth transition probabilities. Cohen’s *d* is used for comparison across studies; inferential tests were conducted on changes in partial Pearson correlations within each study. The “Combined” data point represents the sample-weighted mean effect size across studies. The size of each point represents sample size. Error bars represent 95% CIs.

Results indicated that the conceptual knowledge embodied in ACT-FAST offers a partial explanation for how people accurately predict action transitions. Statistically controlling for proximity on the ACT-FAST dimensions significantly reduced association between participants’ ratings of transition probabilities and the corresponding ground truth estimates in each of studies 1 to 5 (Fig. 3 and table S2). This indicates that people may predict future actions based on their conceptual similarity to current actions. Moreover, it suggests that action concepts themselves may be organized at least in part around the goal of action prediction. However, the specific dimensions that supported this mediation varied across studies (see fig. S1).

## DISCUSSION

The ability to anticipate how others will behave is a cornerstone of human social cognition (*1*). By predicting what someone will do next, one can plan one’s own actions to help, hinder, affiliate with, or avoid them. Much previous research has focused on the perceptible predictors of actions—such as which objects in the environment offer certain action affordance (*4*, *5*)—and the hidden predictors of actions, such as goals, intentions, and emotions (*14*, *15*, *17*). Here, we instead focused on how actions predict themselves. Specifically, we assessed how people capitalize on statistical regularities in action dynamics to make accurate predictions. We found strong evidence that people understand these regularities and use them to make accurate action predictions. Across five preregistered studies, we compared people’s predictions about action transitions with ground truth estimates of the actual transition probabilities between those real-world actions. Results from all five studies indicated that people’s judgments about action transitions were robustly aligned with reality. These predictions were nuanced, capable of predicting both action sequences and clusters of actions that tend to occur close together in time. We also examined how people achieved this accuracy. We found that the structure of people’s action concepts is tuned for action prediction: Combined, the dimensions of the ACT-FAST action taxonomy statistically mediated accuracy in every study. Together, these results indicate that people can accurately predict what others will do next by attending to what others are doing now. The existence of this mechanism for foresight carries wide-ranging implications for understanding how humans interact and coordinate their behavior.

We found evidence for accurate action predictions across four very different ground truth datasets. To measure the actual action transitions, we examined verbs in spoken lines of movie dialog, self-reported daily activities, verbs in goal-directed instruction lists, and hand-annotated actions in videos. These datasets differed in terms of how they define actions—as verbs, broad activities, or precise behaviors. They also differed in their characteristic time scale, from seconds, in the case of the annotated videos, to hours, in the self-reported daily activities. In this way, the present findings complement previous investigation of action prediction that focused on smaller numbers of artificial action transitions (*25*, *28*). The studies differed not only in the actions they considered but also in the populations that supplied the ground truth data. For example, studies 2 and 3 drew upon large nationally representative surveys of the United States (the ATUS), while the AVA dataset used in study 5 featured videos from many different cultures, including excerpts from French, Russian, Chinese, Italian, Polish, Turkish, German, and Thai language films. The diversity of these ground truth datasets helps justify generalizable conclusions from the present results. That said, our reliance on samples of perceivers from Mechanical Turk does constitute an important constraint on generalizability.

The present findings also corroborate the hypothesis that the structure of social knowledge is tuned for prediction. In previous work, we proposed a multilayer model of predictive social cognition (*32*). This model suggests that the mind organizes different domains (layers) of social knowledge into low-dimensional representational “maps.” We previously established that the mind organizes much of its knowledge of actions in accordance with a six-dimensional map, the ACT-FASTaxonomy (*33*). Similarly, a low-dimensional map we have termed the 3d Mind Model accounts for much of the organization of mental state representations (*36*). The dimensions of this mental state map—rationality, social impact, and valence—explain behavioral judgments of mental states, neural activity elicited by thinking about others’ thoughts and feelings, as well as the semantic similarity between state words estimated using text analysis (*37*). On both maps—ACT-FAST and 3d Mind Model—proximity reflects conceptual similarity: Similar actions (e.g., walking and running) and similar states (e.g., happiness and pride) tend to be close together within their respective map. This organization thus offers an efficient way to represent otherwise complex knowledge. A person’s mind need not recall every nuance about every action to effectively understand that action. Rather, it needs only to track an action on six dimensions to maintain a useful conceptual summary of that action (*36*, *37*).

However, the ACT-FAST does more than describe actions; it also predicts them. Placing actions on this map offers an efficient strategy for generating accurate predictions. As we observed in the present investigation, the closer two actions are to each other, the greater the likelihood of transitions between them. Thus, the ACT-FAST map mediates some of the accuracy of people’s action predictions. Similarly, the closer two emotions are in the mental state map of the 3d Mind Model, the more likely one is to transition from one state to the next (*24*). Together, these parallel sets of findings—describing predictive maps of both actions and mental states—support the central thesis of the multilayer model: that the brain creates maps of social domains for the purpose of predicting the future. Although people could represent each and every possible action transition independently, this would be highly inefficient. Representing transitional probabilities via ACT-FAST proximity offers a far more parsimonious way to encode much of the same information. This encoding scheme also permits efficient generalization to newly learned actions: For example, simply by learning that a new action is food related and concrete, one could accurately predict of what actions are likely to precede or follow it.

Although we observed consistent evidence the ACT-FAST dimensions can account for some of the accuracy of action prediction, we must qualify this finding in three ways. First, evidence comes from partial statistical mediation. This mediation explained only a fraction of people’s accuracy (table S2). Moreover, by its nature, this analysis cannot not provide strong evidence for the causal role of the ACT-FAST in action prediction. Experimentally manipulating the availability of conceptual knowledge would provide a causally diagnostic test of its role in this process.

Second, a map like the ACT-FAST cannot account for asymmetric transitions. The model posits that distance on this map predicts transition probabilities. However, distances are always symmetric, whereas transition probabilities are not. We speculate that asymmetric transitions might instead be described in terms of interactions between different layers of social knowledge. For example, the state of hunger might lead to both cooking and eating, which would explain their temporal proximity to each other. But eating might lead to the state of satiation, which would preclude future actions like cooking. Thus, by appealing to the state layer, we can explain why cooking might be unlikely to follow eating, but eating is likely to co-occur or follow cooking.

Third, we found that individual conceptual dimensions did not consistently explain participants’ accuracy across the five studies (fig. S1). That is, although the ACT-FAST as a whole mediated accuracy in each case, different conceptual dimensions contributed to this mediation in each study. We speculate that certain dimensions may characterize action dynamics better at some time scales than at others, or better in some context than in others. For example, the AVA dataset in study 5 featured a much higher temporal resolution than the ATUS dataset used in studies 2 and 3. The food dimension of ACT-FAST mediated accuracy in studies 2 and 3, but not in study 5. This might suggest that food relevance governs action transitions at long time scales (e.g., over which someone might become hungry), but not at short time scales. However, there are many differences between each of the ground truth datasets we draw upon here, making it difficult to infer which specific factors influence the applicability of different conceptual dimensions. Systematically investigating this issue should be a priority for future research.

A more general limitation of the present research is that it focuses exclusively on “offline” action predictions. That is, participants made explicit judgments of the likelihood of decontextualized action transitions, rather than implicit predictions, while embedded in a realistic context. In recent research, we investigated whether the same mechanisms we explore here could also explain these more “online” action predictions (*34*). We found that when people perceive actions in naturalistic stimuli (i.e., while watching an episode of *Sherlock*), their brains spontaneously encode the ACT-FAST coordinates of these actions. Moreover, by representing actions in this way, the brain automatically gains insight into at least the next three actions that are likely to unfold in the video. These results support the present findings and suggest that people can make accurate online—as well as offline—predictions of others’ actions.

In sum, the findings of the present investigation shed light on a foundational human capacity: the ability to predict what someone is going to do next. Although many sources of information might help one formulate such predictions, we find that knowledge of another person’s current action alone is enough to support accurate, nuanced predictions of future actions. We suggest that people can predict others’ actions from scant information because our social knowledge is tuned for prediction. People are highly attuned to statistical regularities that help them predict their environment, so much so that the conceptual structure of actions reflects action dynamics in their environment. These findings have potentially far-reaching implications, from hinting at new ways to measure social impairment to revealing statistical regularities that artificial systems could draw upon to better understand human behavior.

## METHODS

### Open science practices

Data and code from this investigation are freely available on the Open Science Framework (https://osf.io/a5ekv/). Data collection and analysis plans were preregistered in detail for all five studies: study 1 (https://osf.io/k45am/), study 2 (https://osf.io/32n4w/), study 3 (https://osf.io/mtzgp/), study 4 (https://osf.io/ndqtm/), and study 5 (https://osf.io/xsu4z/). We report all measures, manipulations, data exclusions, and deviations from our registered plans.

### Ground truth estimation

To measure the accuracy of predictions about action transitions, it was necessary to first generate a measure of “ground truth”—that is, assess how often action transitions occurred in the world. To this end, we drew on four very different datasets: Study 1 examined verbs in spoken lines of dialog and stage direction in 1036 movie scripts from IMSDb; studies 2 and 3 examined 3.5 million self-reported daily activities from ATUS; study 4 examined verbs in 32,061 instruction sets from WikiHow; study 5 examined actions that were hand-annotated every 3 s in 192 15-min-long videos from the AVA.

#### Study 1: IMSDb

Movies typically describe relatively naturalistic action sequences (see the Supplementary Materials for comparison against study 4 ground truth). The scripts for these movies thus provide a useful window into which action transitions are more or less likely. By operationalizing actions as verbs, we can use movie scripts to derive estimates of the ground truth transition probabilities between actions.

A set of 1036 movie scripts were programmatically downloaded from IMSDb.com as part of an earlier investigation (*33*). Using Python (www.python.org/) and the Natural Language Toolkit (www.nltk.org/), the scripts were split by spoken lines and then tokenized into words. We then searched these words for common verbs, as proxies for actions. Specifically, we identified instances of the 235 most common verb lemmas in WordNet (*38*) as defined by their frequency in the SUBTLEX_US_ corpus (*39*). We then computed the transition probabilities between verbs using an exponential decay model (*40*). For each verb we identified, we counted all other verbs that followed it in the next 50 lines of spoken dialog. For example, if “run” occurred in the current line, and “eat” occurred another 10 lines down the script, we would add to the “run → eat” element of a transition count table. The amount added to this cell of the table was equal to *e*^−*bd*^, where *e* is the base of the natural logarithm, *d* is the distance between verbs in line number, and the weighting factor *b* is a power of 2 ranging from 2^−4^ to 2^3^ by integer exponents. We used *b* = 2^−3^, as this value maximized split-half reliability of the asymmetric component of the transition probability matrices. This formula down-weights verb transitions as an exponential function of the distance between them—in other words, short-range transitions are counted more than long-range transitions. The transition probability matrix was normalized for frequency-based expectations (calculated by multiplying the action frequency vector with its transpose), thereby removing the effects of base rates on transition probabilities (*24*). We took the natural logarithms of the resulting odds, and then *z*-scored the results to produce the final ground truth measure against which to compare participants’ ratings. *Z* scoring was not necessary for the correlational analyses, but was helpful for comparison across datasets (e.g., Fig. 1) and for producing interpretable estimates in the mediational analyses.

We used a simulated annealing algorithm to select an optimal set of 30 actions (out of the 235 common verbs considered) for participants to rate. The verb “to fuck” was manually removed before application of this algorithm to avoid exposing participants to profanity. The simulated annealing algorithm optimized for three criteria. First, it aimed to maximize the reliability of the symmetric component of the transition probability matrix of the selected verbs. Second, it aimed to maximize the reliability of the asymmetric component of the same matrix. Third, it aimed to maximize the association between the ACT-FAST dimensions and the transition probability matrix. This final criterion was measured by regressing the transition probability matrix onto distances between the verbs on each of the ACT-FAST dimensions. These distances were calculated using scores from a text analysis of verb-noun co-occurrences in earlier research (*33*). The minimum regression coefficient was maximized by the selection algorithm. The final set of 30 actions selected by this algorithm is shown in Materials and Methods.

#### Studies 2 and 3: American Time Use Survey

Using verbs as proxies for actions—as we do in studies 1 and 4—has an important drawback: Verbs are polysemous. The same verb (e.g., “running”) can refer to completely different actions (e.g. “running a mile” versus “running an analysis”). The ground truth that we estimate in these studies thus represents a blend of different senses of each verb. To the extent that these blends are misaligned with the meaning people prototypically associated with each verb in isolation, we may observe dampened accuracies in these studies. The ground truth estimates in studies 2 to 3 and 5—which do not rely on verbs—thus provide a useful complement to studies 1 and 4.

In studies 2 and 3, we estimated ground truth using the ATUS. The ATUS is a large, nationally representative survey conducted annually by the U.S. Bureau of Labor Statistics (*35*). Among other measures, each respondent reports on their activities over the course of the previous day. Here, we drew upon these day-recalls for the survey years 2003–2016 (all the years available at the time of data collection). Participants described these actions at multiple levels of granularity, levels defined by the Bureau. In study 2, we focused on broad (“tier 1”) actions. These actions reflect low-granularity descriptions of activities, such as “eating and drinking” or “socializing.” In study 3, we focused on narrow (“tier 2”) actions. These actions reflect more detailed, granular descriptions of actions, for example, dividing “sports” into “participation in” versus “attending an event.” We removed (i) actions in a catchall “unable to code” category (as indicated by the ATUS survey administrators; see coding manuals: https://www.bls.gov/tus/lexicons.htm), (ii) tier 2 actions that occurred, on average, less than once for every 10 years’ worth of recorded days, and (iii) participants who reported only a single action all day. Together, this totaled approximately 3.5 million actions reported by 181,335 participants in study 2. In study 3, we additionally eliminated (iv) all travel-related activities (these activities were overly specific, in that they specified the next activity to which the person was traveling), (v) all unspecified miscellaneous activities, and (iv) all activities performed by less than 1 in 30 participants on average. Miscellaneous activities are those that are specified at one of the ATUS’s three levels of granularity, but not at a lower level. In this case, an activity might be identified at level 1 (the lowest granularity, used in study 2) as “Personal Care Activities.” However, at level 2, it might not fall into any of the existing subcategories (sleeping, grooming, health-related self-care, etc.) and might thus fall into the miscellaneous subcategory for Personal Care Activities. Because it is difficult to interpret the meaning of these miscellaneous categories, we excluded such items in study 3. Together, this left a total of approximately 2.7 million actions reported by 181,333 participants, across a final set of 29 activities at the tier 2 level in study 3. See the Supplementary Materials for complete lists of activities in both studies.

In each study, we computed transition probability matrices between activities (before steps 4 to 6 above). We computed these transition probabilities using an exponential weighting system, in which the weight assigned to the transition from one activity to a later activity was *e*^−(*d*−1)^, where *e* is the base of the natural logarithm and *d* is the number of activities between each pair of actions. As with the IMSDb data, these transition probabilities were then normalized by frequency-based expectations to remove base rates, log-transformed, and finally *z*-scored to produce the final set of ground truth estimates against which to compare participants’ ratings.

#### Study 4: WikiHow

WikiHow is a website that contains tens of thousands of sets of instructions for how to achieve particular goals in a series of steps. The goals range widely, from “How to Resize Text with HTML and Javascript” to “How to Prune Cilantro.” These articles offer a wide range of normative action sequence. The “rvest” package in R (*41*) was used to retrieve 32,061 pages of instructions distributed across all major categories on WikiHow.com. Not all WikiHow.com articles are listed under the major category headings; however, we used all articles that were so listed at the time of the scrape.

We calculated action transitions by examining verbs present in consecutive steps of the instructions. We first divided the text of each page into steps and then tokenized the words within each step. Within each step, we then searched for any of 192 verbs that have previously been rated on the ACT-FAST dimensions as part of study 1, or in earlier research (*33*). Transitions were counted between verbs in consecutive steps of each page. Low-frequency verbs—with fewer than 10 transitions to each other action—were removed sequentially. The counts were then normalized by frequency-based expectations to remove base rates, log-transformed, and *z*-scored.

Within the 192 × 192 transition probability matrix, we selected an optimized set of 100 action transitions to present to participants. A simulated annealing algorithm was used to select the optimal set according to five criteria: (i) maximize the mean correlation between transition probabilities and ACT-FAST ratings; (ii) maximize the minimum correlation between transition probabilities and ACT-FAST ratings; (iii) maximize the mean partial correlation between transition probabilities and ACT-FAST ratings; (iv) maximize the minimum partial correlation between transition probabilities and ACT-FAST ratings; (v) and minimize mean absolute correlation between the ACT-FAST ratings themselves. By selecting according to these criteria, we chose the best possible set of action for detecting mediation of the action prediction by the ACT-FAST dimensions. To avoid overfitting, we performed this optimization procedure using a transition probability matrix computed using one-half of the data. The accuracy and dimensional mediation of participants’ ratings were subsequently tested against the other half. We also did not test whether the ground truth estimates from WikiHow were correlated with ACT-FAST proximity because they were selected for just this property. Instead, we only tested the association between ACT-FAST and transitions ratings, and whether ACT-FAST statistically mediates the association between rated and ground truth transitions.

#### Study 5: Google’s Atomic Video Actions

The final dataset contains action sequences present in video clips. The AVA dataset consisted of 192 15-min-long videos curated by Google. The actions in these videos were hand-annotated every 3 s (https://research.google.com/ava/). Using these annotations for 80 actions, we computed the transition probabilities. Specifically, we counted action transitions from one 3-s segment of video to the next. The resulting 80 × 80 matrix was highly sparse—that is, there were many cells that contained zero transitions across the entire dataset. Although these transitions are clearly unlikely, the small number of transitions also makes their precise estimate unstable. We removed all sparse cells by iteratively removing the action with the largest number of empty cells in the transition probability matrix. This yielded a final set of 23 actions (see the Supplementary Materials). The transition counts between these actions were normalized by frequency-based expectations, log-transformed, and *z*-scored to produce the final ground truth against which we compared participants’ judgments.

An unintentional deviation from our registered plan for study 5 occurred due to a bug in the code used to select actions. As a result of this bug, we initially collected transition probability ratings with respect to an arbitrary set of 23 actions, rather than the 23 actions actually selected by the procedure described above. We analyzed these data in accordance with our registered plan, at which point we found the error. We then reran the experiment with the intended actions. The former results are reported as study 5′ in the Supplementary Materials, while the latter are reported as study 5 above. Despite the coding error, study 5 and study 5′ produced qualitatively identical results. Thus, this deviation and its subsequent correction do not alter the interpretation of the main results, except perhaps to strengthen them via additional evidence.

### Participants

Participants were recruited via Amazon Mechanical Turk and TurkPrime (*42*) for all five studies in this investigation. All participants provided informed consent in a manner approved by the Institutional Review Board of Princeton University. For each study, we collected two separate samples of participants. The first sample completed a transition probability task, in which they provided ratings of the transition probabilities between actions; the second sample completed an action dimension task, in which they provided ratings of the same set of actions on the six ACT-FAST dimensions. The data used to estimate the ground truth transition probabilities were all publicly available.

Target sample sizes were determined via a priori power analyses. The target sample size for the transition probability tasks was determined based on the effect sizes observed in a previous study of similar design focused on emotion transitions (*24*). These effects were very large, varying between *d* = 1.92 and 2.58. However, participants in those studies made a larger number of ratings than we planned to collect in the present investigation. We expected that this might attenuate effect sizes, and thus, we assumed that the effect in the present studies might be correspondingly smaller (*d* = 0.476). A parametric power analysis indicated that a sample of 60 participants would be adequate to provide 95% power for this effect size. However, out of an abundance of caution, we doubled this to 120. Studies 1 to 3 and 5 all targeted this number. In study 4, we only collected data on the transition probability, with action dimension ratings instead drawn from previous data collections. As such, for this study, we targeted *N* = 160 to match the maximum targeted sample size for the action dimension task.

In study 1, we targeted a sample size of 50 participants for the dimension rating sample. Based on previous data (*33*), we estimated that this would provide an interparticipant Cronbach’s α > 0.9 for all six dimensions. Based on the results of study 1, we estimated that the overall effect size of the dimensional mediation was *d* = 0.35. A parametric power analysis indicated that a sample size of 157 would be necessary to achieve familywise power of 95% for the six ACT-FAST dimensions. We thus targeted a sample size of 160 for the action dimension sample in study 2. Some of the subsequent studies indicated larger mediation effects, but we nonetheless retained the target of *N* = 160 for studies 3 and 5.

Participants were excluded based on two preregistered criteria: indicating that they were neither native nor fluent English speakers, or providing 10 or fewer unique responses on in the rating task. These criteria led to the exclusion of a total of 27 participants across nine samples, a rate of 2%. See Table 4 for a breakdown of participant exclusions and the demographics of the retained participants.

**Table 4:** Participant sample sizes, exclusions, and demographics.

**Study**	**Task**	***N*_1_**	**Lang.**	**Unique**	***N*_F_**	**Female**	**Male**	**Other**	**Mean age**	**Age range**
1	Transitions	118	2	0	116	50	66	0/0	35.04	20–65
1	Dimensions	50	0	1	49	25	24	0/0	33.96	22–56
2	Transitions	123	1	1	121	57	62	2/0	36.53	20–66
2	Dimensions	159	1	3	155	74	80	1/0	35.14	20–68
3	Transitions	120	1	2	117	54	63	0/0	34.74	21–68
3	Dimensions	157	0	5	152	63	89	0/0	36.51	21–68
4	Transitions	158	0	2	156	81	75	0/0	37.62	20–71
5	Transitions	119	1	1	117	49	68	0/0	35.32	21–63
5	Dimensions	159	1	5	153	70	81	1/1	37.31	19–71

Note: *N*_1_ indicates the number of participants who completed each survey. Lang. indicates participants who were excluded for indicating that they were nonnative, nonfluent English speakers. Unique indicates participants who were excluded for providing 10 or fewer unique responses in the task. *N*_F_ indicates the final sample size for each survey. The Other column indicates participants who did not indicate their gender as female or male. The first value indicates “Prefer not to answer,” while the second indicates “Other.”

### Transition probability task

Our primary measure was participants’ action transition predictions. We used these predictions to test whether people had accurate perceptions of how actions transitioned from one to the next—as assessed by our ground truth datasets. In each study, participants were linked from TurkPrime to a Qualtrics-based survey. On each trial, participants rated the transition probability between one action and another. The actions varied in each study (see the Supplementary Materials). In study 1, each participant rated a random subset of 120 out of 900 total transitions between 30 actions. In study 2, each participant rated all 289 transitions between 17 actions. In study 3, each participant rated a random subset of 120 out of 841 total transitions between 29 actions. In study 4, each participant rated all of an optimized set of 100 transitions. In study 5, each participant rated a random subset of 100 of 529 total transitions between 23 actions. In each case, action transitions were presented in different random order to each participant. After the transition ratings, participants provided demographic information.

### Action dimension task

Separate groups of participants rated actions on the dimensions of the ACT-FASTaxonomy. These ratings allowed us to test whether the participants’ accurate action transition ratings were mediated by conceptual knowledge of those actions. The actions (verbs) used in study 4 had already been rated—some in study 1 here, and others in a separate investigation (*33*). Participants in this task were linked from TurkPrime to a Qualtrics-based survey. On each trial, participants rated an action on one of the six ACT-FAST dimensions: Abstraction/Sociality, Creation, Tradition, Food, Animacy, and Spiritualism. For example, a participant might rate the action “to sell” on the spiritualism dimension with the following prompt: “Please rate the action below on the psychological dimension of **work** versus **worship**. **Work** actions tend to be related to effort, business, money, and management. **Worship** actions tend to be used in the context of religion, poetry or metaphor, and spirituality.” The prompts and definitions for each dimension were validated as part of an earlier investigation (*33*) and are listed in the Supplementary Materials. Participants made ratings on a continuous line scale, anchored per the definition for each dimension. Participants rated all actions in a given study on all six dimensions. Ratings were grouped into blocks by dimension. Blocks were randomized for each participant. Action order was randomized within each block.

### Statistical analysis

#### Measuring accuracy

To measure the accuracy of participants’ action transition predictions, we correlated their action transition ratings with the corresponding ground truth estimates. These correlations were carried out at two levels: at the individual level and the item level.

At the individual level, we Spearman-correlated each participant’s ratings with the corresponding ground truth estimates. This procedure produced a ρ coefficient for each participant. These coefficients were normalized using Fisher’s *r*-to-*z* transformation to allow them to be interpreted as a linear interval variable. We then assessed average individual accuracy by comparing the mean *z*-transformed ρ value against zero. Two statistical tests were used to assess the statistical significance of the difference between the average ρ and zero: a parametric one-sample *t* test and a percentile bootstrap. The two tests agreed in all cases. Standardized effect sizes (Cohen’s *d*s) were calculated for the individual accuracy effect by taking the mean *z*-transformed ρ and dividing it by the SD of the *z*-transformed ρ’s.

In addition to testing participant accuracy at the individual level, we also tested at the item level, by correlating ground truth with transition ratings averaged across all participants. The primary analysis (Fig. 1) was carried out with respect to the full transition probability matrix between actions. In all but study 4, three additional variants of the item analysis were carried out to assess the robustness and nuance of perceiver accuracy (table S1). The first variant considered only the off-diagonal elements of the matrix (i.e., no transitions from one action back to itself). This helps determines whether participants’ accuracy could be explained away by a reliance on the intuition that actions are autocorrelated. The second variant examined the symmetric component of the transition probability matrix. That is, participants’ ratings were averaged across the diagonal, the ground truth transitions were likewise averaged across the diagonal, and then the results were correlated with each other. This indicates whether accuracy relies entirely upon knowledge of action sequences (e.g., as in an instruction manual) in which each action must strictly be undertaken after the previous one. The final variant examined only the asymmetric component of the transition probability matrix. This indicates whether participants have knowledge of action sequences, or other cases in which action A was likely to precede action B, but B was not likely to precede A.

In each variant of the item analysis, Spearman correlations were used to estimate the association between the (vectorized) rating and ground truth matrices. The Mantel test was used to assess the statistical significance of these correlations. The Mantel test is a permutation method that recognizes the dependencies implicit in distance/similarity matrices. The elements of such matrices are not independent of one another, and so the Mantel test instead randomizes the rows and columns of the matrices instead. Calculating the correlations between these randomized matrices generates an empirical null distribution that can be used to compute appropriately conservative *P* values. No inferential item analyses were conducted in study 4 due to the intentionally incomplete transition probability matrices examined in that study.

To better contextualize the effects observed in the item-level analyses, we estimated both the correlations that would be expected due to chance, and the maximum correlation that could be expected given the reliability of the data. To estimate the chance correlations, we computed the medians of the empirical null distributions generated by the Mantel test. These values were very close to zero for the “no diagonal” and “asymmetric” variants, but slightly elevated for the “full-matrix” and “symmetric” variants (table S1).

To estimate the maximum correlation that we could expect to observe in each study, we conducted a post hoc noise ceiling analysis. This analysis started by calculating the split-half reliability of each transition rating dataset for studies 1 to 3 and 5 (study 4 was excluded because we did not conduct inferential item analyses in this study). We then estimated the corresponding reliabilities of the ground truth transitions by correlating the upper and lower triangular components of these matrices. Following Spearman’s method for correlation disattenuation, we multiplied these reliabilities together and then took the square root of their product. The resulting values indicate the highest correlations that could be expected due to chance in each study. We have included these values in Table 2 to better contextualize the item analysis results.

It was not possible to conduct the four variants of the item analyses at the individual level in most studies because each participant only rated a random subset of action transitions. For example, we could not examine asymmetries at the individual level because a given participant was unlikely to have rated both directions of transition between many pairs of actions. However, due to the small number of actions in study 2, each participant in that study rated the entire set of action transitions. This afforded us the opportunity to test the various analysis variants at the individual level as well.

#### Conceptual mediation

Here, we tested the hypothesis that conceptual similarity, reflected in proximity on the ACT-FAST dimensions, supports the accuracy of participants’ predictions of action transitions. To test the statistical mediation of accuracy by ACT-FAST, we conducted a partial correlation analysis. First, we computed the partial Pearson correlation between transition ratings and ground truth (symmetric off-diagonal components only), controlling for proximity on all six dimensions. Proximity was defined as the negative absolute difference between ratings of each action on a given dimension. We then subtracted the full partial correlations from the corresponding zero-order correlation to determine how much the strength of the association had changed as a function of controlling for the ACT-FAST proximities. This process was repeated for each participant in the dimension rating study, yielding a set of differences in *z*-transformed *r* values. The statistical significance of this change in correlation was tested against zero using one-sample *t* tests and percentile bootstrapping. We also repeated this analysis at the level of individual dimensions (fig. S1), comparing the partial correlation controlling for all six dimensions to the partial correlation controlling for all but one (in turn) to isolate the unique mediational contribution of each component of the ACT-FAST.

The mediation analyses described above were carried out slightly differently in study 4, due to its design. Like study 1, study 4 used verbs as proxies for actions. To reduce the need for redundant data collection, in study 4, we used verbs that had already been rated. Some of these ratings were provided in study 1, and the rest were provided in a previous investigation (*33*). As a result, the partial correlation analysis featured separate regressions for each participant in the transition rating sample, with each participants’ transition ratings predicted by proximities based on the single set of average dimension ratings.

## SUPPLEMENTARY MATERIALS

Supplementary material for this article is available at http://advances.sciencemag.org/cgi/content/full/7/9/eabd4995/DC1
